# Intermediate filaments: Integration of cell mechanical properties during migration

**DOI:** 10.3389/fcell.2022.951816

**Published:** 2022-08-05

**Authors:** Elvira Infante, Sandrine Etienne-Manneville

**Affiliations:** Cell Polarity, Migration and Cancer Unit, Institut Pasteur, UMR3691 CNRS, Université Paris-Cité, Equipe Labellisée Ligue Contre le Cancer, Paris, France

**Keywords:** vimentin (intermediate filaments), cell adhesion, cell migration, mechanotransduction, cytoskeleton, cytoskeletal crosstalk, cell mechanics

## Abstract

Cell migration is a vital and dynamic process required for the development of multicellular organisms and for immune system responses, tissue renewal and wound healing in adults. It also contributes to a variety of human diseases such as cancers, autoimmune diseases, chronic inflammation and fibrosis. The cytoskeleton, which includes actin microfilaments, microtubules, and intermediate filaments (IFs), is responsible for the maintenance of animal cell shape and structural integrity. Each cytoskeletal network contributes its unique properties to dynamic cell behaviour, such as cell polarization, membrane protrusion, cell adhesion and contraction. Hence, cell migration requires the dynamic orchestration of all cytoskeleton components. Among these, IFs have emerged as a molecular scaffold with unique mechanical features and a key player in the cell resilience to mechanical stresses during migration through complex 3D environment. Moreover, accumulating evidence illustrates the participation of IFs in signalling cascades and cytoskeletal crosstalk. Teaming up with actin and microtubules, IFs contribute to the active generation of forces required for cell adhesion and mesenchymal migration and invasion. Here we summarize and discuss how IFs integrate mechanical properties and signalling functions to control cell migration in a wide spectrum of physiological and pathological situations.

## Introduction

Although the process of migration varies with the cell type, the cell environment and the molecular cues inducing and controlling migration, the cytoskeleton always is at the heart of the machinery promoting cell movement. Cell migration initially requires structural, morphological and functional polarization of the cell along a front-to-rear axis which defines the direction of movement. The different elements of the cytoskeleton, actin microfilaments and stress fibers, microtubules and intermediate filaments organize along this polarity axis. The polarization of the cytoskeletal networks relies on multiple signalling pathways triggered by soluble factors, like chemoattractant gradients, or by cell adhesion to the extracellular matrix (ECM) and/or to other cells (Mayor and Etienne-Manneville, 2016). Once polarized the cytoskeleton stabilizes cell polarity, promotes membrane protrusion at the front, controls the dynamics of cell adhesive structures from the front to the back so that cells can adhere to the substrate, contract and retract while also interacting with their neighbours (Lense and Etienne-Manneville, 2015). The amoeboid mode of migration is characterised by a low adhesion to the extracellular matrix (ECM) together with a generally high acto-myosin contractility, which generates pushing forces at the cell rear propelling membrane protrusions or blebs at the front and forward cell movement (Graziani et al., 2022). In contrast, mesenchymal migration requires integrin-mediated focal adhesions (FAs) which mediate cell adhesion to the ECM (Kanchanawong et al., 2010; De Pascalis and Etienne-Manneville, 2017). Contractile acto-myosin cables anchored at focal adhesions exert traction forces that pull the cell forward. Not only actin, but also microtubules and IFs interact with FAs and contribute to FA dynamics and FA signalling. During mesenchymal migration, the microtubule network aligns along the front-to-rear axis, while the microtubule-organizing centre and the Golgi apparatus often reposition in front of the nucleus (Elric and Etienne-Manneville, 2014; Jimenez et al., 2021). Similarly, the IF network is reorganized. It frequently accumulates in a perinuclear region where it is pushed by the actin retrograde flow (Dupin et al., 2011) and at the same time it extends towards the cell front following microtubule tracks (Sakamoto et al., 2013; Leduc and Etienne-Manneville, 2017). Microtubules and then IFs reach FAs where they directly or indirectly interact with mechanosensing molecules, signalling components and the actin cytoskeleton (Seetharaman and Etienne-Manneville, 2019). Microtubules have recently been shown to participate directly in mechanotransduction and mechanosensitive cell migration (De Pascalis and Etienne-Manneville, 2017; Seetharaman and Etienne-Manneville, 2019), which has recently raised the question of IF’s contribution to these processes.

Cell migration not only requires the active and dynamically controlled generation of forces, it also requires the cell resilience to deformations. This aspect of cell mechanics is most critical when cells navigate in a complex 3D environment, composed of the ECM entangled components and neighbouring cells. In this context, the three cytoskeletal networks form distinct, yet connected, structural scaffolds with different mechanical properties which together define the visco-elastic properties of the cell. In contrast to actin and microtubules, IFs’ contribution does not rely on the energy-consuming activity of molecular motors but instead on the unique mechanical properties of the filaments and the network. Actin filaments, microtubules, and IFs all contribute to cell resilience under small deformations. However, both F-actin and microtubules yield or disassemble under moderate strains (Janmey et al., 1991). It is thus the IF network connected to cellular organelles and structures which protects the cell from mechanical damage under large strains. Moreover, the function of IFs goes far beyond that of a purely mechanical support. IFs also act as scaffolds for signaling molecules and serve as a stress buffer, operating as a phosphate sponge in stressed cells (Pallari and Eriksson, 2006). IFs’ functions in cell signalling are essential during wound healing by safeguarding the recruitment and targeting of signaling molecules, while in the vasculature, vimentin IFs were shown to tune the Notch signaling pathway and arterial remodeling in response to shear stress (van Engeland et al., 2019). In this review, we will summarize the recent findings showing how IFs can contribute both their mechanical and signalling properties to cell migration and invasion.

## Intermediate filaments in the intrinsic mechanical properties of invading cells

### Structure and mechanical properties of intermediate filaments

Unlike, microtubules and F-actin, IFs can assemble *in vitro* without any cofactors or nucleoside triphosphates, through coiled-coil interactions mediated by the central rod domains which are a common characteristics all IF proteins (Chernyatina et al., 2015). Mature IFs are non-polar structures (Robert et al., 2016) with a diameter of 10 nm, intermediate between actin filaments (∼8 nm) and microtubules (∼25 nm). In cells, IF proteins can also be found as soluble tetramers and also as small 50 nm filaments, called ULFs (Unit Length Filaments), formed by the lateral association of the tetramers. The ULFs can assemble in small filaments called squiggles or in longer filaments that can reach several μm in length and spread throughout the cytoplasm. In vertebrates, IF proteins are classified into six subtypes, based on their sequence homology (Dutour-Provenzano and Etienne-Manneville, 2021). In contrast to the ubiquitously expressed nuclear lamins, cytoplasmic IF proteins display a cell-type specific expression pattern and form filaments whose composition and organization change during embryogenesis, development, and pathogenesis. Whether these variations in IF composition are directly involved in the distinct motile behaviour of various cell types is an interesting hypothesis to explore.

The mechanical properties of IFs are very different from those of actin filaments or microtubules (Sapra and Medalia, 2021; van Bodegraven and Etienne-Manneville, 2021). Single IFs are particularly flexible as shown by their short persistence length observed both *in vitro* and in cells. However, this flexibility varies with the composition of the filaments, and more particularly with the length and the charge density of the side chains of IF proteins (Beck et al., 2010). Neurofilaments display different persistence length, due to a different ratio between light and heavy chains (van Bodegraven and Etienne-Manneville, 2021).

Nuclear and cytoplasmic IFs are also highly stretchable. Depending on the experimental setting, desmin, vimentin, keratin or lamins IFs can be stretched by 240%–300% before breaking (Sapra and Medalia, 2021). IFs are not only very elastic; they also show a strain-stiffening response. IF proteins have the unique feature of undergoing molecular structural changes in response to external loads (Ackbarow et al., 2009). The elasticity observed at low strains results, for a large part, from the elastic stretching of the coiled-coil α-helical domains of the IF proteins (Block et al., 2017). Higher extension of the α-helical domains induces additional changes in conformation ultimately leading to β-sheet structures. At higher strains IFs stiffen. This strain-stiffening corresponds to the more difficult extension of the β-sheets. The α-helix to β-sheet transition of cytoplasmic vimentin was recently demonstrated at the cellular level by protein vibrational microscopy, where cellular tension resulted in conformational changes of vimentin within cells suggesting that the *in vitro* observation of IFs mechanics are likely to occur *in vivo* (Fleissner et al., 2020). Another fascinating property of IFs is their loading-rate dependent mechanical response. Because of that, IFs are often metaphorically compared to safety belt. Fast stretching can induce stiffening of the filaments at 50% strain, while at low velocity, IFs do not stiffen until they are stretched to about 200% (Block et al., 2017). The mechanical properties of IF networks involve the intrinsic single molecule mechanics and also the crosslinking between individual IF molecules. When the stress is so high that it leads to the rupture of IF network, a softening is observed. The softening of the network is transient and appears essentially due to the loss of interaction between filaments (Aufderhorst-Roberts and Koenderink, 2019). The mechanical stability of IFs depends on the amino acids composition of IF proteins as shown by comparing keratin and vimentin. The rod and tail domains of keratins are enriched in hydrophobic residues required for their crosslinking. In contrast, vimentin network assembly involves electrostatic interactions with negatively charged amino acids. These specific mechanical properties direct their capability to resist compression, stretching and bending forces, and provide a mechanical support adapted to the various cell-types and tissues. The wide variety of IF molecular composition is likely to provide a wide spectrum of mechanical properties. Moreover, IF diversity can also provide a large panoply of molecular interactions. How IF physical interactions within the IF network and also with the other cytoskeletal components contribute to the visco-elastic properties of the cells and can be adapted to the cell environment still remains to be fully elucidated.

### Intermediate filaments contribution to mechanics of migrating cells

**FIGURE 1:**
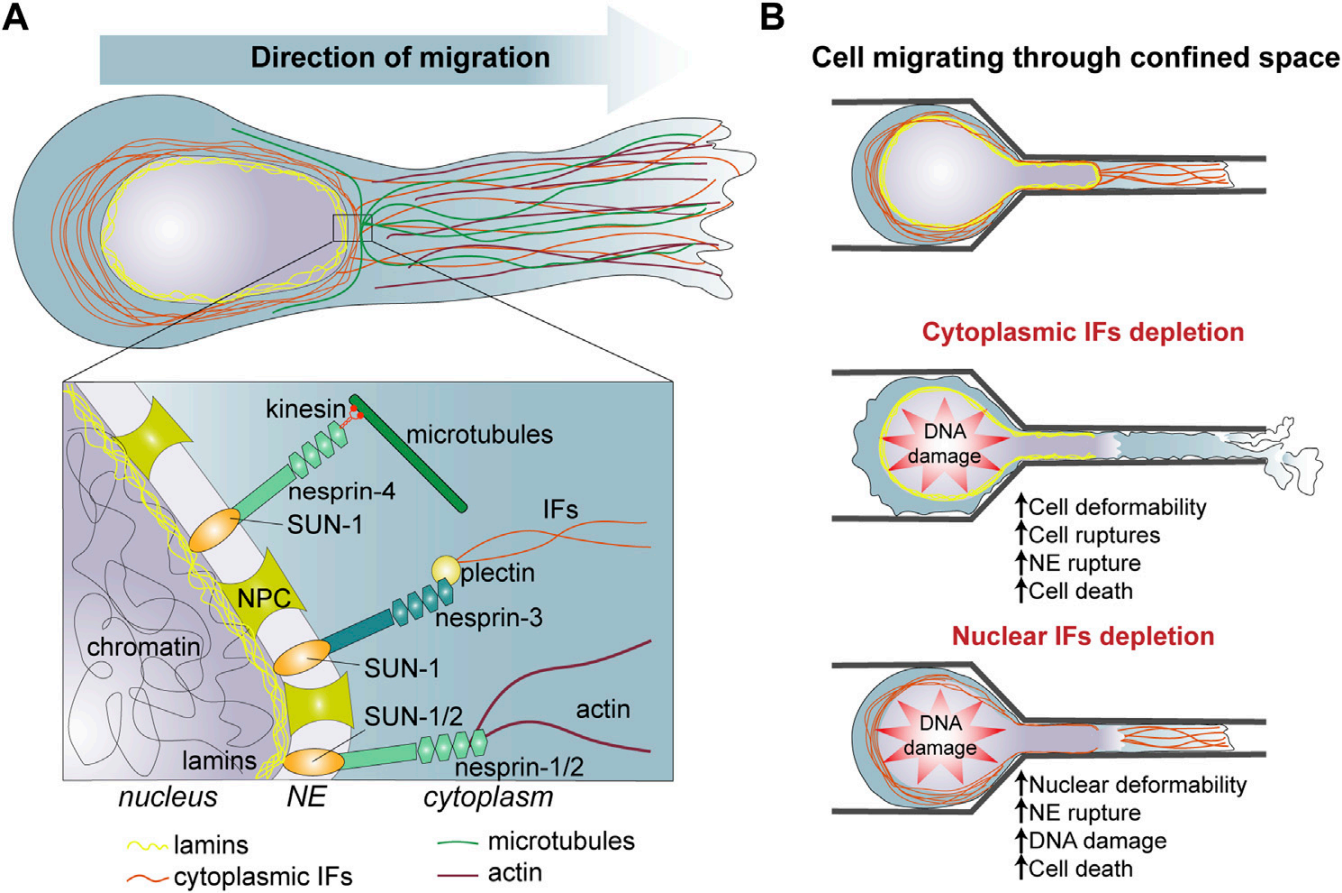
**(A)** Organization of intermediate filaments and interaction with other nuclear and cytoplasmic components in migrating cells. **(B)** Role of intermediate filaments in cells migrating through confined space. [**(B)**, Top] Cells migrating into a complex 3D environment undergo squeezing and deformation of the cytoplasm and the nucleus. [**(B)**, Middle] Cytoplasmic IFs protect the cell from strong cell deformation, nuclear deformation and NE rupture which often leads to DNA damage and cell death. [**(B)**, Bottom] Nuclear IFs protect the nucleus from constricting forces responsible of large nuclear deformation, NE rupture, DNA damage and cell death. IFs, intermediate filaments; NE, nuclear envelope; NPC, nuclear pore complex.

The role of IF in the intrinsic mechanical properties of cells can directly impact cell invasive properties. During invasion of surrounding tissues, cells can experience severe deformations. While the structural integrity of eukaryotic cells under small deformations involves actin filaments, microtubules, and IFs, the IF networks dominate the cytoplasmic mechanics and maintain cell viability under large deformations (Figure 1). Numerous studies probing intracellularly or extracellularly cell mechanical properties have shown that depletion of keratin, vimentin and desmin decreases cell stiffness [for a review, see (Sapra and Medalia, 2021; van Bodegraven and Etienne-Manneville, 2021)]. For instance, single cell nanomechanics experiments using AFM, magnetic and optical tweezers demonstrated that the deletion of type I or type II keratins decreases the Young’s modulus of keratinocytes by more than 50% (Seltmann et al., 2013a; Ramms et al., 2013) (Table 1). In contrast, the overexpression of desmin or vimentin causes a cell stiffening (Charrier et al., 2018). More recently, vimentin IFs were shown to determine cell resilience (Hu et al., 2019). IFs appear mainly responsible of the cytoplasm elasticity while actin and microtubules contribute to viscosity and the interaction between the three cytoskeletal components is needed to resist large forces. The combination of a hyperelastic IF network with quickly recoverable cytoskeletal components forms a mechanically robust structure which can recover after damage. By interacting with cytoskeletal components and cytoplasmic organelles, the vimentin network effectively disperses local deformations in the cytoplasm and slows down the viscoelastic relaxation, protecting organelles against mechanical damage. When embedded in hydrogel and submitted to stretching to recapitulate the mechanical stress experienced during tissue invasion, vimentin-depleted fibroblasts are less viable than control cells (Hu et al., 2019) (Table 1). However, human mesenchymal stem cells knocked down for vimentin and embedded in 4% agarose hydrogels are more resistant to compression, suggesting that compression may involve additional mechanical cell responses such as actin filaments and microtubules resistance to the mechanical load (Sharma et al., 2017) (Table 1).

**TABLE 1:** Specific roles of intermediate filament proteins.

Type	Proteins	Cell mechanics	Mechanotransduction	Migration
I-II	Keratins	↑ Cell stiffness (Seltmann et al., 2013a; Ramms et al., 2013; Laly et al., 2021) ↑ Cell resilience (Seltmann et al., 2013a) ↓ Traction forces (Wang et al., 2020) ↑ Stress fibers (Fujiwara et al., 2016; Wang et al., 2020)	↑ Nuclear mechanotransduction (Laly et al., 2021) ↑ Mechanosensitive cell responses (Weber et al., 2012; Mariani et al., 2020; Laly et al., 2021)	↑ Cell-cell adhesion (Jacob et al., 2018; Wang et al., 2018) ↑ Cell-BM adhesion (Wang et al., 2018; Wang et al., 2020) ↓ Cell migration (Weber et al., 2012; Seltmann et al., 2013a; Seltmann et al., 2013b)
III	Vimentin GFAP Desmin	↑ Cell stiffness (Brown et al., 2001; Rathje et al., 2014; Charrier et al., 2018) ↑ Cell resilience (Sharma et al., 2017; Hu et al., 2019) ↑ Cell viability (Hu et al., 2019) ↓ Nucleus deformation (Patteson et al., 2019) ↓ NE rupture (Patteson et al., 2019) ↑ Traction forces (De Pascalis et al., 2018) ↑ Stress fibers (Jiu et al., 2015; Jiu et al., 2017; De Pascalis et al., 2018) ↑ Cell stiffness (Charrier et al., 2018)	↑ Mechanosensitive cell responses (Swoger et al., 2020)	↑ FAs lifetime (De Pascalis et al., 2018) ↑ Cell adhesion (Ivaska et al., 2005; Havel et al., 2015; Kim et al., 2016) ↑ Cell migration (Helfand et al., 2011; Zhu et al., 2011; De Pascalis et al., 2018; Trogden et al., 2018; Lavenus et al., 2020; Ratnayake et al., 2021) ↑ Cell invasion (Zhu et al., 2011; Messica et al., 2017; Ratnayake et al., 2021) ↑ FAs turnover (De Pascalis et al., 2018)
V	Lamin A/C	↑ Cell stiffness (Lee et al., 2007) ↑ Nucleus stiffness (Dahl et al., 2004; Harada et al., 2014; Wintner et al., 2020) ↑ Nuclear viscosity (Wintner et al., 2020) ↓ NE rupture (Morelli et al., 2017; Earle et al., 2020) ↑ Cell viability (55, 62)	↑ Mechanosensitive cell responses (Swift et al., 2013; Urciuoli et al., 2021) ↑ Nuclear mechanotransduction (Swift et al., 2013)	↓ Cell migration (Lee et al., 2007; Urciuoli et al., 2021) ↓ Migration through confined space (Harada et al., 2014)
	Lamin B1	↑ Nucleus elasticity (Wintner et al., 2020)	↑ Mechanosensitive cell responses (Swift et al., 2013)	
VI	Nestin	↑ Traction forces (De Pascalis et al., 2018) ↑ Stress fibers (De Pascalis et al., 2018)		↑ FAs lifetime (Hyder et al., 2014; De Pascalis et al., 2018) ↑ Cell migration (De Pascalis et al., 2018) ↑ Cell invasion (Hyder et al., 2014)

The table shows how each type of intermediate filament proteins impacts on cell mechanics, mechanotransduction and cell migration. FA, focal adhesions; NE, nuclear envelope. ↑ (resp. ↓) indicates that IF protein expression induces an increase (resp. decrease) in cell mechanics, mechanotransduction or migration.

The mechanical functions of the IF network have several consequences in terms of cell migratory and invasive capacities (Figure 1B). Vimentin expression increase the migratory properties of the highly invasive breast carcinoma cell line MDA-MB-231 when the cells are densely packed whereas it does not have much effect on the random migration of sparse cells (Messica et al., 2017), suggesting that cell density causes mechanical stresses which are best supported in presence of vimentin. In the context of cancer progression, vimentin-associated cell stiffness may facilitate the survival and the invasion of cell groups. Vimentin also promotes cell viability by enhancing cell stretchability and mechanical resilience. This may reveal crucial when cells experience shear stress as they travel through the bloodstream. However, in the case of migration through tight spaces, the ability of single cells to deform is essential and the absence of IFs which decreases cell stiffness would then facilitate cell movement. Accordingly, keratin depletion strongly promotes epithelial cell migration through the small pores of a Boyden chamber. However, this increased migration also resulted in a high number of cell ruptures. While passing through the pores, keratin-depleted cells left remnants of cell behind them, again supporting the role of IFs in supporting cell resilience to deformation (Seltmann et al., 2013a) (Figure 1B). Yet, during cancer cell invasion, cells can alleviate the mechanical stress exerted by the ECM thanks to a metalloproteinase machinery that allows the degradation of the ECM (Denais et al., 2016; Ferrari et al., 2019). It would be interesting to explore a potential correlation between metalloproteinase expression and IF composition in various cancer cells.

**FIGURE 2:**
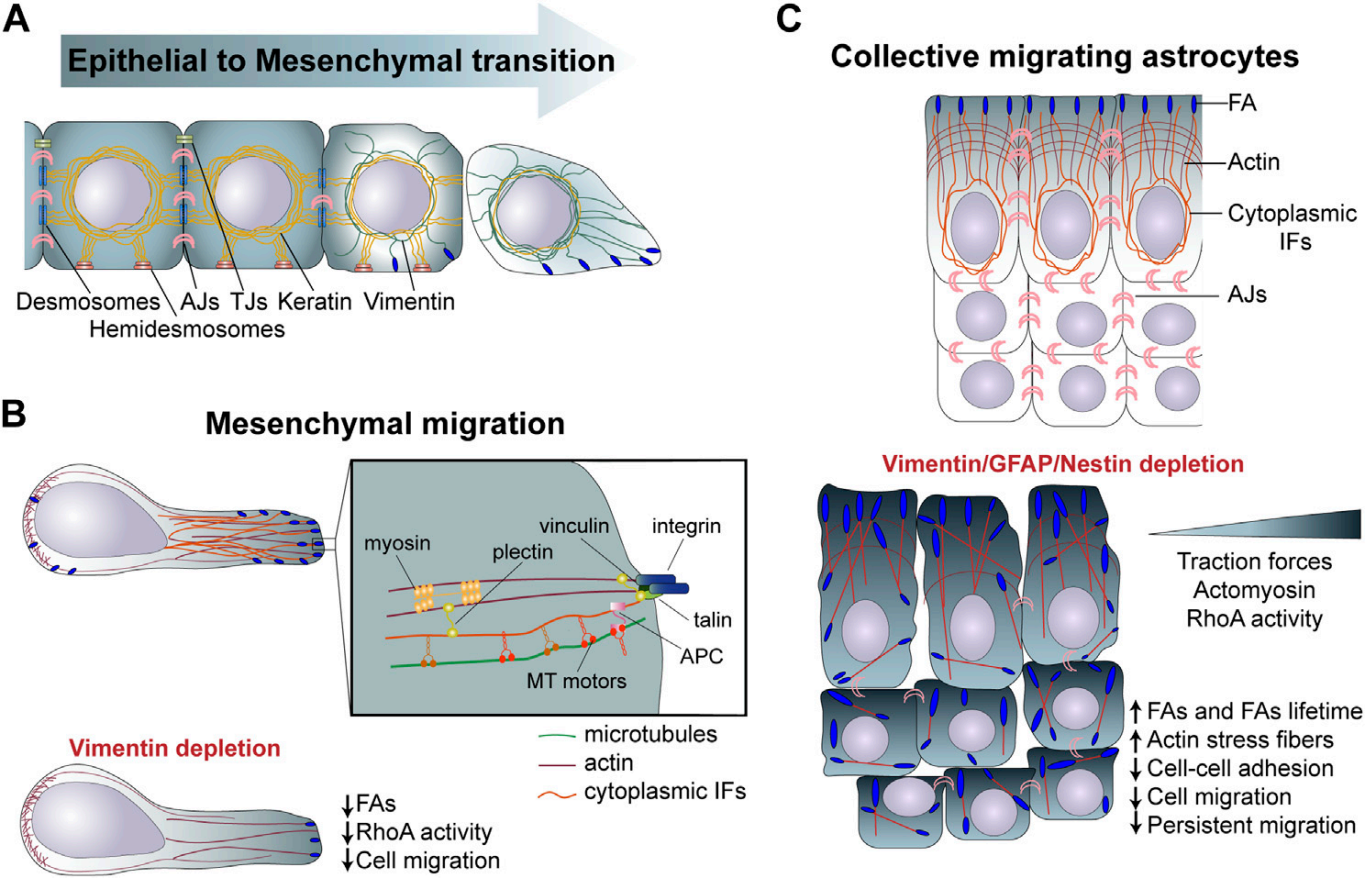
**(A)** Role of intermediate filaments in EMT. During EMT cells are gradually decreasing the expression of keratin, following an increase in vimentin expression promoting cell invasion. **(B)** During mesenchymal migration cells attach to the extracellular matrix through FAs, which link, via the cytoskeletan linker plectin, to IFs. Vimentin depletion reduces FAs structure and cell motility. **(C)** In collectively migrating astrocytes cytoplasmic IFs interact actomyosin machinery altogether mastering cell migration. [**(C)**, Bottom] Depletion of GFAP, vimentin and nestin affects interjunctional transverse arcs and increases stress fibers. The increase in FAs lifetime and in RhoA activity and tranction forces disrupts collective astrocyte migration, speed and directionality. IFs, intermediate filaments; FAs, Focal adhesions; EMT, epithelial to mesenchymal transition; MT, microtubules; TJs, Tight junctions.

Given the specific mechanical properties of IFs and the specificity of each IF proteins, the modification of IF composition affects cell mechanical properties and, thereby, the cell migratory behavior. During EMT, vimentin is upregulated as keratins are progressively downregulated. Possibly linked to their distinct mechanical properties, vimentin IFs primarily provide resistance to stress and compression at the single-cell level, unlike the keratin network, which supports cell-cell adhesion and tissue-level resistance to mechanical stress and compression (Jacob et al., 2018) (Figure 2A, Table 1). Another level of complexity results from the constant remodelling of the IF network within motile cells (Robert et al., 2016). Endothelial cells exposed to shear stress show a rapid redistribution of vimentin IFs (Helmke et al., 2000). IF are especially remodelled above the nucleus and in proximity to cell-cell contacts, suggesting a redistribution of intracellular forces in response to shear stress. In circulating peripheral blood T lymphocytes, vimentin is the main IFs responsible of cell rigidity. Upon polarisation vimentin retracts and concentrates close to the uropod. This collapse is required for cell deformability during transendothelial migration (Brown et al., 2001) (Table 1). This suggest the existence of local regulatory mechanisms which affect the IF network organization and maybe also composition to locally adapt the cell mechanical properties.

The structural reorganisation of the network is certainly indicative of a change in filament crosslinking and interactions with cellular structures. Post-translational modifications such as phosphorylation, glycosylation and, less commonly, sumoylation, palmytoylation, citrullination and acetylation can control the dynamics and the molecular interactions within and between the IF network and other cellular structures. To this date, phosphorylation is the best characterized regulator of IF assembly and organization (Snider and Omary, 2014). The formation of tetramers and ULFs is regulated by phosphorylation of residues in the head domain of IF proteins (Kornreich et al., 2015). On the vimentin for instance, Ser6, Ser7, Ser8, Ser33, Ser39, Ser56, Ser71, Ser72, and Ser83 on the head regions have been shown to undergo phosphorylation and multiple kinases have been involved such as Protein kinase A (PKA), PKC, cyclin-dependent kinase 1 (CDK1), Ca^2+^/calmodulin-dependent protein kinase II (CaM kinase II), RhoA kinase, RAC-alpha serine/threonine-protein kinase (AKT1) and RAF proto-oncogene serine/threonine-protein kinases (Raf-1-associated kinases) (Yasui et al., 2001; Ratnayake et al., 2017; Ratnayake et al., 2018). Phosphorylation of IF proteins is a fast and reversible way to regulate IF solubility and thereby affect the resistance as well as the resilience of the network. During cell migration, phosphorylation of keratin 8 has been shown to induce or inhibit cell migration depending on the cell type. Vimentin phosphorylation at Ser39 by AKT promotes cell migration and control vimentin catalytic cleavage (Zhu et al., 2011). Recently, acetylation of vimentin at Lys120 has also been associated with an increase in cell migration [for a review on IF post-translational modifications, please see (Snider and Omary, 2014; MacTaggart and Kashina, 2021) and reference therein]. More indirect regulatory mechanisms may also affect the organization and functions of IFs. For instance, the oncogene expression of simian virus 40 large T antigen, c-Myc, and cyclin E, through the upregulation of the tubulin deacetylase histone deacetylase 6 (HDAC6) and by modifying the spatial distribution of acetylated microtubules, can lead to a reorganization of the vimentin network and to an increase in cell stiffness (Rathje et al., 2014).

In conclusion, IFs form a molecular scaffold essential for the intrinsic mechanical properties of cells. Our more recent understanding of the specificities of each IF proteins together with their ability to form complex heteropolymers strongly suggest that regulation of the IF composition and post-translational modifications of IF proteins may allow the cells to adapt, their mechanical properties to their environment on a short or a long time scale, and as a consequence to regulate their motile behaviour.

### Intermediate filaments and the protection of the nucleus

While the cytoplasmic IFs are essential for the resilience to cytoplasmic deformation, nuclear IFs also participate in cell invasion by protecting the nucleus against mechanical stresses (Figure 1B). Indeed, the plasma membrane and the cytoplasm are relatively deformable and able to pass through spaces of less than 1 μm in diameter, but the nucleus, the largest organelle of the cell, is the limiting factor during migration through tight spaces (McGregor et al., 2016; Estabrook et al., 2021). As cells progresses in 3D confined environment, compression and traction forces deform the nucleus. This can eventually cause nuclear blebbing. Nuclear blebs lack nuclear envelop (NE) proteins and lamina and can contain chromatin herniation. Under prolonged external or internal—partially generated by actomyosin contraction forces—pressures, nuclear blebs may result in NE rupture and eventually DNA damage due to the action of cytoplasmic proteins or mechanical forces (Lim et al., 2016; Shah et al., 2017).

The nuclear lamins participates in the nuclear mechanical properties. Cryo-electron tomography of lamina meshwork by Turgay et al., revealed the structure and organisation of nuclear lamina in mammalian nuclei. The lamin meshwork is composed of ∼3.5 nm thick filaments accumulated at the nuclear periphery and underneath the nuclear pores (Turgay et al., 2017). The mechanical properties of *in situ* assembled lamin filaments and networks have been characterised in isolated nuclei by AFM, cryo-electron tomography, and molecular dynamics. This study revealed that lamin filaments can reversibly deform at low force regime (< 500 pN) by acting as a shock absorber and can also withstand constant forces up to 2 nN. These properties may be necessary to prevent filaments breakage and network failure (Sapra et al., 2020). The nuclear lamina limits the deformability of the nucleus (Calero-Cuenca et al., 2018). Up to 3 µm deformations, the elastic resistance of the nucleus is determined by chromatin, but the resistance to larger deformations is mainly dictated by lamins (Dahl et al., 2004; Stephens et al., 2019). Lamin A/C plays a key role in controlling nuclear stiffness (Ho and Lammerding, 2012) and increases cell resilience to large nuclear deformations (Cupesi et al., 2010) (Table 1). More recently, Wintner et al. showed that both lamin A and lamin B1 participate nuclear elasticity but that lamin A is the major contributor to nuclear viscosity (Wintner et al., 2020). Interestingly, lamin A/C expression increases nuclear stiffness and is correlated to the mechanical properties of the tissue, so that nuclear mechanics scales with the extracellular stiffness (Swift et al., 2013; Cho et al., 2017), suggesting a possible feedback mechanism by which cells could adapt the composition of the protective envelop of their genetic material to the physical properties of their environment. Cancer cells with more deformable nuclei have higher rates of migration through small constrictions. Highly metastatic breast cancer cells express low level of lamin A/C, leading to more malleable nuclei and promoting their migration through confined environments (Hsia et al., 2021). High metastatic osteosarcoma cells, a type of bone cancer that metastasize into the lungs, also express low levels of Lamin A/C and spread on soft substrate resembling lung parenchyma (Urciuoli et al., 2021).

Lamin IFs are not only guardians of the NE integrity, they also protect the genomic DNA from damages caused by mechanical constraints (Wolf et al., 2013) (Figure 1B, Table 1). Mouse models of striated muscle laminopathies nicely illustrate how lamin mutations increase the deformability of the nucleus, give rise to extensive NE ruptures and DNA damage and finally can cause the death of skeletal muscle cells. The NE rupture seems to be independent by actomyosin contractility, but instead linked to the nuclear movement mediated by Kif5b-microtubule motors (Earle et al., 2020). The chaperone protein HSPB2 colocalises in nuclear foci with lamin A, and behave as liquid droplets. The aberrant phase separation of HSPB2 modifies Lamin A and chromatin distribution affecting nuclear function and integrity (Morelli et al., 2017). While cells migrate through constrictions the size of constrain inversely correlates with the number of NE breaks. Depletion of lamin A/C and B2 significantly increases the numbers of NE ruptures (Denais et al., 2016; Raab et al., 2016). These NE ruptures expose nuclear DNA to the cytoplasmic exonuclease TREX1 resulting in DNA damage and genomic alterations (Nader et al., 2021). Cells that express high level of nuclear lamin A/C have a decrease capability to squeeze through confined spaces but nuclear stiffness can favour genomic stability and cancer cell survival (Harada et al., 2014).

Additionally, cytoplasmic IFs seem to participate in the protection of the nucleus. In 3D collagen gels and during migration through small channels vimentin-null mouse embryonic fibroblasts display larger nuclear deformations than control cells (Figure 1B). There are also more prone to NE ruptures and DNA damage, supporting the survival function of IFs during migration in confined environment (Patteson et al., 2019). The upregulation of vimentin observed in pathological conditions such as EMT may contribute in preventing cell death and promoting tumour cell invasion. Perinuclear IFs interact with the NE via the LINC (Linker of Nucleoskeleton and Cytoskeleton) complex, using in particular Nesprin 3’s ability to interact with plectin (Chang et al., 2015). This interaction is involved in the control of nuclear localization and nuclear shape during cell migration. On the internal side of the NE, the LINC complex interacts with the nuclear lamina through the Sun proteins, creating a molecular bridge between cytoplasmic and nuclear IFs (Figure 1A). Expression of KASH domain which perturbs Sun-Nesprin interaction, impacts nuclear mechanics similarly to the deletion of lamin A. Conversely, both the LINC complex and lamin A influence the cytoplasmic cytoskeleton and cell migration showing the interplay between cytoplasmic and nuclear cytoskeleton (Lee et al., 2007; Hall, 2009) (Table 1). The cytosplasmic and nuclear IFs form two distinct but connected networks which contribute to cell and nuclear resistance and, more importantly, resilience in face of the mechanical challenges encountered during invasion of dense, complex 3D environment. The crosstalk between these two IF networks still need to be investigated in detail and may reveal how cells integrate their cortical, cytoplasmic and nuclear mechanics to adapt to the properties of their environment and the cell motile behaviour.

## Intermediate filaments in active mechanical cell responses of migrating cells

IFs not only serve as a structural scaffold governing cellular mechanics, they also contribute to signalling cascades and thereby influence cell proliferation, cell death, cell differentiation as well as cell adhesion, and motility [(Cheng et al., 2016), for review (Etienne-Manneville, 2018)]. During cell invasion, the signalling functions of IFs play a crucial role in the regulation of the migration machinery, including cell adhesion to the ECM, actin dynamics and acto-myosin contractility, microtubule-driven cell polarity and cell-cell interactions involved in collective migration.

### Intermediate filaments are involved in cytoskeletal crosstalk

IFs physically and biochemically connect with the other cytoskeletal components, actin and microtubules, and these interactions are essential for cell migration.

Vimentin IFs decrease the diffusion of actin monomers suggesting a specific cytoplasmic interaction between actin and vimentin which might also influence cell mechanics (Wu et al., 2022). In the case of mesenchymal migration, the cell extends a protrusion. Actin polymerizes close to the plasma membrane to form lamellipodia in response to the activity of small GTPase Rac at the cell leading edge. In parallel Rac induces the phosphorylation of vimentin on Ser-38 which causes the disassembly of the filaments at the cell periphery promoting actin-driven membrane protrusion (Helfand et al., 2011). Interestingly, using a phosphoproteomic screen in lung cancer cells, the Rac1 guanine nucleotide exchange factor (GEF) VAV2 was identified as a downstream target of vimentin which induces VAV2 phosphorylation and the localization to FAs where it activates Rac1 (Havel et al., 2015). Similarly AKT, downstream of the PI3K, can interact with and phosphorylate vimentin Ser38 to induce motility and invasion (Zhu et al., 2011) (Table 1). In human prostate cancer cells, vimentin is phosphorylated at Ser33, Ser39 and Ser56 by atypical PKCs at the cell front, leading to the local disassembly of vimentin IFs, lamellipodium formation and cell migration (Ratnayake et al., 2021).

The interplay between IFs and microtubules also contribute to the coordinated regulation of the different cytoskeletal components. *In vitro* single molecule interaction experiments, using optical tweezers, have demonstrated that vimentin prevents microtubule depolymerization (Schaedel et al., 2021). Intriguingly, the transport of vimentin, neurofilaments and peripherin depends on their interaction with microtubules and microtubules motors, dynein and kinesins, whereas keratins particles travel along F-actin (Robert et al., 2016). Vimentin and keratin short filaments and particles cycle in a centripetal motion which is based on actin dynamics from the cell periphery, where they are elongated in long filaments and assembled into a network, to the perinuclear area (Martys et al., 1999; Windoffer et al., 2004). Surprisingly, the long mature filaments of keratins 8/18 and vimentin may also use another way of transport mediated by the KIF5B isoform of the microtubules motor kinesin-1 (Robert et al., 2018).

IFs crosstalk with microtubules is at play in migratory cells. As microtubules reach focal adhesions at the front and become polarized along the front-to-rear axis, they contribute to cell polarization (Etienne-Manneville, 2013). This includes the polarization of the vimentin-containing IF network. IFs are preferentially transported by kinesin-mediated transport along microtubules which are growing towards the cell front; the IF network elongate along the polarity axis (Leduc and Etienne-Manneville, 2017). Moreover, microtubule-associated APC (Adenomatous Polyposis Coli) interacts with vimentin filaments and promote their polymerization (Sakamoto et al., 2013). Finally, the two networks are tightly interacting via cytoskeletal crosslinkers such as plectin, which can form bridges between IFs and both microtubules and actin microfilaments (Figure 2A). This probably promotes or stabilize the parallel organization of the cytoskeletal networks along the polarity axis (Wiche et al., 2015a). Indeed, at the cell front microtubules undergo dynamic instability with a short grow-shrink cycle of 3–5 min, vimentin IFs, which are much less dynamic with a grow-shrink cycle of more than 10 min, have been proposed to act as a template for the re-polymerization of tubulin, stabilizing the direction of microtubule growth and cell polarity and promoting persistent directed migration (Gan et al., 2016). In endothelial cell, the efficient crosstalk between microtubules and IFs network entails the direct association with the BCAS3 domain of the cytoskeletal protein Rudhira. This interaction enhances MT stability (acetyl and Glu-tubulin) and insure cell migration during angiogenesis (Joshi and Inamdar, 2019).

The properties and the regulation of the cell cytoskeleton is not a simple addition of the properties of each cytoskeletal network but in fact results from an intricate crosstalk in which IFs, actin and microtubules combine their unique properties. Understanding how direct and indirect interactions between the various networks influence cell mechanical responses remains a challenge.

### Intermediate filaments regulate cell adhesion to the extracellular matrix

As the IF network polarizes, IFs reach FAs, where they have been shown to interact directly and indirectly via the cytoskeletal linker plectin with core FA proteins such as integrins or talin (Wiche et al., 2015b; Leube et al., 2015) (Figure 2). Vimentin- or plectin-deficient fibroblasts show an altered FA turnover and directional migration (Gregor et al., 2014). In migrating astrocytes depletion of the glial IFs GFAP, vimentin, and nestin or depletion of the cytoskeletal linker, plectin, increases the number of FAs in leaders cells and also strongly affects their distribution. IFs depletion also reduces the FA turnover and the generation of traction forces, ultimately affecting cell speed and direction of collective migration (De Pascalis et al., 2018) (Figure 2, Table 1). IFs can contribute to the regulation of FA dynamics in different ways. First, nestin and vimentin modulate integrin expression and trafficking to the PM. In nestin-depleted prostate cancer cells α5 and β1 integrins are more active and targeted to the cell membrane, which promote FAK-dependent matrix degradation and cancer cell invasion (Hyder et al., 2014). Moreover, the head domain of vimentin was found to bind the cytoplasmic tail of β3 integrin, controlling cell adhesion and migration. Vimentin filaments underneath the PM can directly interact and increase β3 integrin avidity, resulting in integrin clustering and adhesion (Kim et al., 2016). This vimentin-integrin interaction supports breast cancer cell migration and *in vivo* lung metastasis formation. Since PTM influences assembly of IFs into filaments, inhibition of protein kinase C (PKC)-mediated phosphorylation of vimentin leads to the trapping of integrins into vesicles, preventing integrin trafficking, FA adhesion turnover and cell motility (Ivaska et al., 2005). Finally, The Rac1 GEF VAV2, when activated by vimentin promotes stabilization of FAs and adhesion (Havel et al., 2015).

At the cell cortex, super-resolution images and cryo-electron tomography of mouse embryonic fibroblasts revealed that vimentin IF and F-actin form an interpenetrating network. The IFs appear to be incorporated into actin fibers at the time of stress fiber formation, generating a strong synergistic interaction. This association of IFs with actin stress fibers as also been observed at the front of migrating astrocytes in proximity to FAs (Seetharaman et al., 2022). The vimentin/F-actin network delays G-actin diffusion, enhances contraction forces and eventually impacts cell contractility (Wu et al., 2022). Considering the mechanical properties of IFs and their connection with both FAs and the actin cytoskeleton, it would not be surprising to find that IFs participate in mechanotransduction at FAs. Recently the contribution of keratin in mechano-responses has been demonstrated by the group of Connelly (Laly et al., 2021). Keratinocytes exposed to different degrees of matrix stiffness adapt by the formation of a rigid meshwork of keratin bundles, which is less deformable, and an overall increase in cell stiffness. The role of vimentin in mechanosensing was recently studied by Patteson AE group by designing hydrogels with controlled elastic and viscoelastic material properties. Cells lacking vimentin showed an impaired spreading on viscous substrates (Swoger et al., 2020) (Table 1). Changes in substrate viscoelasticity, but not in substrate elasticity, led to a reorganisation of vimentin filaments into a mesh-like cage in the perinuclear area, which may indirectly affect mechanosensitive nuclear responses. This type of studies should certainly be done in other cell types to determine if all IF proteins have similar role in mechanotransduction at FAs or if changes in IF composition may affect how cells respond to the mechanical properties of the substrate.

In epithelial cells, keratin IFs have been known to associate with other adhesive and tensional-sensing structures, known as hemidesmosomes, which strongly enhance the adhesion of epithelial cells with lamin-containing basement membranes. Hemidesmosomes are composed by integrin α6β4, which interact with the plectin, which interacts with IFs. The plectin-IFs interaction is regulated by C-terminus phosphorylation of plectin at the S4642 residue (Wang et al., 2020) (Figure 2A, Table 1). The altered distribution of hemidesmosomes in KO keratin increases adhesion and migration of keratinocytes compared to wild-type cells, consistent with the downregulation of keratin observed during epithelial-mesenchymal transition (Seltmann et al., 2013b). Moreover, hemidesmosomes promote the redistribution of αVβ5 from FAs to clathrin lattices by reducing cellular tension (Wang et al., 2020). In keratinocytes and carcinoma cell lines, hemidesmosomes participate in the regulation of cellular tension and traction forces. This requires an intact laminin-integrin β4-plectin-keratin linkage, which decreases the capability of cells to spread and create mature FAs, and consequently to exert traction forces on the substratum (Wang et al., 2020).

### Cytoplasmic intermediate filaments influence acto-myosin contractililty and mechanotransduction

IFs influence FAs not only via the control of membrane trafficking and integrin signalling but also via their impact on force-dependent maturation of FAs (Jiu et al., 2015; van Bodegraven and Etienne-Manneville, 2021). In cells depleted of vimentin, actin stress fiber assembly and contractility are increased, a property that is reversed upon re-expression of vimentin (Jiu et al., 2017) (Figure 2). Vimentin regulates actin assembly and contraction by downregulating the phosphorylation of GEF-H1, a component of the RhoA pathway involved in actin reorganization (Jiu et al., 2017). The molecular mechanisms involved in the IF-mediated regulation of GEF-H1 is still unknown. It has been speculated that vimentin acts as a “phosphorylation sink.” As vimentin is heavily phosphorylated by multiple kinases, it may reduce the net activity of these kinases on other substrates such as GEF-H1. This phosphorylation sink could also interfere with the phosphorylation of actin, leading to an increased instability of actin filaments. In addition to its effect on GEF-H1, Vimentin can also induce the phosphorylation of the RhoA-GEF, ARHGEF2, and, in this case, promote RhoA activity and contractility (Jiu et al., 2017).

Keratin also, in particular keratin 18 activates RhoA via the RhoA-GEF Solo in epithelial cells (Fujiwara et al., 2016). Moreover keratin 6a/b interacts directly with myosin IIA. This stabilizes myosin IIA, increases traction forces and slows down migration (Wang et al., 2018). The mechanoresponse of keratinocytes to matrix stiffness depends on an efficient crosstalk between IFs and acto-myosin. Incrementing matrix stiffness leads to an increase in lamin A/C expression, which is normally implicated in chromatin remodeling and gene expression (Swift et al., 2013). However, two different types of dominant mutations in the basal keratin K5 or K14, which cause epidermolysis bullosa simplex, inhibit the increase of lamin A/C in response to substrate stiffness. On the contrary, the total loss of type I keratin and plectin induce the up-regulation of F-actin stress fibers and lamin A/C, implying that distinct alteration of the keratin network can differently affect mechanotransduction (Laly et al., 2021).

The importance of IF and acto-myosin crosstalk is best exemplified during confined migration, where the mechanical properties of IFs combines with their impact on cytoskeletal regulation During amoeboid migration, accumulation of vimentin in the cell body can counteract the actomyosin force produced by leader blebs, decreasing the speed of migration (Lavenus et al., 2020). Indeed, the cholesterol-lowering drug simvastatin, which induces vimentin bundling (Trogden et al., 2018), inhibits ameboid migration in confined space (Lavenus et al., 2020). These results suggest that the bundling of vimentin limits cell compressibility and consequently, the flow of cytoplasm to the front of the cell required for ameboid migration. The role of IFs in orchestrating the mechanics of cell migration in confined environment was demonstrated further by Petri et al. When ECM proteolysis is inhibited but a robust cell-matrix adhesion is maintained, mesenchymal cells rely on a pressurised cytoplasm at the front of the nucleus for cell migration. This mechanism, described as the “nuclear piston” requires vimentin and its interaction via plectin with the NE protein Nesprin 3. Together they transmit mechanical forces from the cytoskeleton to the nucleus. Actomyosin contractility and the vimentin–nesprin-3 complex is required to generate forces that can pull the nucleus and compartmentalise hydrostatic pressure between the nucleus and the leading edge, supporting migration in a 3D environment (Petrie et al., 2014). Overall, the direct and indirect interactions between IFs and actin not only influence the generation of forces exerted on the substrate to propel the cell forward but they also contribute to the transmission of forces within the cell and transmit these forces to the nucleus, helping the movement of this large organelle as the cell passes through tight spaces.

### Intermediate filaments at cell-cell contacts and their functions in collective migration

The IF network interacts with multiple structures in cells to form a connecting molecular scaffold, which contribute to tissue integrity and, in the case of motile cells, plays an important role in collective migration, by participating in the mechanocoupling between neighbouring cells. Although the nature of the interactions between IFs and adherens junctions remains to be fully characterized, experiments on migrating monolayers of astrocytes showed that IFs control the distribution of forces at adherens junctions between leader cells and prevent the accumulation of forces at the center of the monolayer. Depleting IFs or plectin, severely affects traction forces, a mechanism via which IFs control cell-cell interaction and master collective cell migration (De Pascalis et al., 2018) (Figure 2C, Table 1).

The interaction of keratin IFs with the epithelial desmosomes is better characterized and involves plakins. This connection is a key regulator of epidermal structure and function, in which the polarization of different layers maintain the epidermal barrier. In epithelia, the apical surface exerts an actomyosin-dependent tension which is critical for the regulation of tissue homeostasis. Disruption of desmosome-IF connection by the overexpression of the dominant negative form of desmoplakin causes a decrease in cell-cell compression forces and promotes substrate adhesion (Broussard et al., 2017). This alteration in cell-cell mechanocoupling impairs epithelial polarization and stratification during early morphogenesis (Broussard et al., 2021). Previous work from Weber and DeSimone on collective cell migration of Xenopus mesendoderm, has demonstrated that application of tension through a magnetic tweezer to the desmosomal receptor C-cadherin triggers the localization of keratin network to stressed cell-cell boundary (Weber et al., 2012) (Table 1). Targeting of the keratin network at cell-cell contacts requires 14-3-3 proteins—which are already know to bind and control the activity of different IFs—and contribute to the organization of mechanosensitive cell-cell contacts, with a possible impact on coordinated cell migration (Mariani et al., 2020) (Table 1).

## Concluding remarks and future perspectives

Due to their structure and polymerization properties, IFs have unique mechanical properties, which can vary depending on the composition of the IF filaments. IF composition can be regulated via the regulation of gene expression or by post-translational modifications to give rise a wide variety of modulable networks whose mechanical properties define, for a large part, cell mechanics. However, IFs do not act alone and the different elements of the cytoskeleton combined with their interactions with numerous cellular structures and organelles result in a material with unique, complex mechanical properties and confer cell resilience to mechanical challenges.

The complex interplay between cytoskeletal networks is based on molecular interactions via cytoskeletal linkers and also on the role of each network, including IFs, in cell signalling. Although not discuss here, one must to not forget that IFs does not only influence acto-myosin contractility, actin and microtubule dynamics, microtubule-driven transport but they more generally participate in the regulation of cell survival, cell proliferation, cell death. How the mechanical properties of specific IF proteins and corresponding IF network influence cell mechanics, simultaneously with intracellular signalling to control cell migration and invasion requires further investigation. Nevertheless, recent evidence point to a strong impact of IF composition on cell migratory behaviour. For instance, Keratin 14, was identified in ovarian cancer metastases as a marker of invasive and migrating potential (Bilandzic et al., 2019). In the context of breast cancer, keratin 14 is also highly expressed in cells leading collective invasion *in vitro* and *in vivo*. Keratin 14 depletion disrupts collective invasion (Cheung et al., 2013). Changes in the tumour microenvironment, such as chemokine gradients and mechanical cues can trigger the polarisation of keratin14 positive cells to the leading edge in breast cancer organoids model (Hwang et al., 2019). How changes in IF composition and IF network mechanical properties can modify cell ability to proliferate, die or differentiate in response to the physical properties of the environment must be further studied to better understand whether the expression pattern of IF proteins may be more than a marker of cell differentiation, a direct participant in the control of cell behaviour. Alterations of IF polymerization and organization are implicated in severe pathological processes such as skin abnormalities, cardiomyopathy and cancer. Determining whether these alterations solely affect the mechanical properties of the IF network or if perturbation of the IF networks results in profound defects in cell ability to actively respond to their surrounding could contribute in the design of new therapies to treat these diseases.
